# Effect of *Echium amoenum* Fisch. et Mey a Traditional Iranian Herbal Remedy in an Experimental Model of Acute Pancreatitis

**DOI:** 10.5402/2012/141548

**Published:** 2012-09-13

**Authors:** Alireza Abed, Mohsen Minaiyan, Alireza Ghannadi, Parvin Mahzouni, Mohammad Reza Babavalian

**Affiliations:** ^1^Department of Pharmacology and Toxicology and Isfahan Pharmaceutical Sciences Research Center, School of Pharmacy and Pharmaceutical Sciences, Isfahan University of Medical Sciences, Isfahan 8146-73461, Iran; ^2^Department of Pharmacognosy and Isfahan Pharmaceutical Sciences Research Center, School of Pharmacy and Pharmaceutical Sciences, Isfahan University of Medical Sciences, Isfahan 8146-73461, Iran; ^3^Department of Clinical Pathology, School of Medicine, Isfahan University of Medical Sciences, Isfahan 8146-73461, Iran; ^4^Islamic Azad University, Qazvin Branch, Qazvin 8146-73461, Iran

## Abstract

Acute pancreatitis is a morbid inflammatory condition of pancreas with limited specific therapy. Enhanced oxidative stress plays an important role in induction and progression of acute pancreatitis. So reducing oxidative stress may relieve this pathogenic process. *Echium amoenum* Fisch. and Mey has been implemented in Iranian folk medicine for several centuries. Antioxidant, analgesic, immunomodulatory, and anxiolytic properties of *E. amoenum* suggest that this plant may have beneficial effects in the management of acute pancreatitis. The aim of this study was to evaluate the protective effect of petals of *E. amoenum* extract (EAE) on a murine model of pancreatitis. Acute pancreatitis was induced by five intraperitoneal (i.p.) injection of cerulein (50 **μ**g/kg) with 1h intervals which was characterized by pancreatic inflammation and increase in the serum level of digestive enzymes, in comparison to normal mice. EAE (100, 200, and 400 mg/kg) was administered i.p., 30 minutes before induction of pancreatitis. Pretreatment with EAE (400 mg/kg) reduced significantly the inflammatory response of cerulein-induced acute pancreatitis by ameliorating pancreatic edema, amylase and lipase serum levels, proinflammatory cytokines, myeloperoxidase activity, lipid peroxidation and pathological alteration. These results show that EAE attenuates the severity of cerulein-induced acute pancreatitis with an anti-inflammatory, immunomodulatory and antioxidant effects.

## 1. Introduction

Acute pancreatitis is an inflammatory condition of pancreas which is characterized by increased serum level of digestive enzymes, sudden onset, high mortality rate, and multiple organ failure characteristics [1]. Circulatory shock, cardiac insufficiency, renal, respiratory, and hepatic failure are the main causes of death [2]. Alcohol beverages drinking and biliary tract disorders are the most important etiologies of pancreatitis; however, other factors including viral infections such as mumps and hepatitis type A&B, and drugs such as tetracyclines, furosemide, and estrogens as well as hypertriglyceridemia and hypercalcemia are also involved [3]. Oxidative stress like hydrogen peroxide (H_2_O_2_), superoxide and hydroxyl radicals, and proinflammatory cytokines such as tumor necrosis factor (TNF)-alpha have been shown to induce acinar cell injury and to be involved in the progression of this illness from acinar cell injuries to a fetal systemic reactions, where oxygen free radicals and lipid peroxidation play important roles in the development of pancreatic inflammation [4].

Among several experimental models of acute pancreatitis that are similar to human pancreatitis, cerulein-induced acute pancreatitis is one of the best known and widely used experimental models [5]. Indeed, immune-derived cytokines as well as free oxygen radicals act as main inflammatory mediators in this model so it was used in current study.

*Echium amoenum* (*E. amoenum*) Fisch. and Mey (Boraginaceae), an important Iranian traditional remedy, is widely used as a tonic, tranquillizer, diaphoretic, and as a remedy for cough, sore throat, and pneumonia [6]. It is believed that this plant possesses antibacterial, antioxidant, analgesic, anxiolytic, antidepressant and immunomodulatory properties [7–12]. Also it has been shown that *E. amoenum* aqueous extract was effective in the treatment of obsessive-compulsive disorder [13]. Dried violet-blue petals of *E. amoenum* have been recently recognized as an important source of phenolic compounds like rosmarinic acid, cyaniding, and delphinidin [14]. 

Cyanidin 3-glucoside, the most common anthocyanin, which is present in petals of *E. amoenum* attenuates PGE2 production and cyclooxygenase-2 expressions by inhibiting activation and translocation of c-Jun and NF-*κ*B factors into nucleus [15]. Also the neuroprotective activity of cyanidin 3-glucoside has been investigated by Min et al. They suggested that the beneficial effect was related to attenuation of brain superoxide levels resulted from blocking apoptosis-inducing factor release in mitochondria [16]. 

Delphinidin, the other anthocyanin which is present in petals of *E. amoenum*, inhibits TNF-alpha induced COX-2 expression by directly inhibiting Fyn kinase activity [17]. Rosmarinic acid which is present in *E. amoenum* has also antimicrobial, antiviral, and anti-inflammatory effects [18]. Moreover, the protective activity of rosmarinic acid from *Perilla frutescens* on liver injury was examined by Osakabe et al. The results showed that hepatoprotective effect of rosmarinic acid is due to the scavenging or reducing superoxide or peroxynitrite free radicals [19]. The present study was performed to evaluate the protective effects of petals of *E. amoenum* hydroalcoholic extract (EAE) in a murine model of acute pancreatitis which caused by cerulein administration. To gain access to better insight into the mechanism(s) of action of the observed anti-inflammatory effects of EAE on pancreatitis, we have investigated the effects of EAE on pancreatic edema, leukocyte infiltration, amylase and lipase level, TNF-alpha, interleukin 6 (IL-6), myeloperoxidase activity, and lipid peroxidation. 

## 2. Materials and Methods

### 2.1. Plant Material and Extraction

*E. amoenum* petals (flowers) were collected from Ghazvin, Iran in summer, and authenticated by Professor Mohammadreza Rahiminejad, Isfahan University, Isfahan, Iran. The voucher specimen of *E. amoenum* was deposited in the Herbarium Department of our school with number 1147.

For preparation of hydroalcoholic extract, dried and finely powdered petals (50 g) were soaked in adequate volume of ethanol : water (70 : 30), and the extraction was carried out for 48 h to obtain full extract using percolator apparatus. The product was then shaken, filtered, and evaporated in a rotary evaporator to obtain semisolid extract under reduced pressure and then freeze-dried [20].

### 2.2. Determination of Anthocyanins

Anthocyanins have a critical role in the color quality of many plants. With a change in pH, anthocyanin pigments undergo reversible structural conversions. Colored oxonium form prevails at pH 1.0; however, the colorless hemiketal form exists at pH 4.5. The difference in absorbance of oxonium and hemiketal form at *λ* max of 510 nm is related to the anthocyanin content.

Anthocyanin concentration was calculated as cyanidin-3-glucoside equivalent by the following equation:

(1) Anthocyanin  content  as  cyanidin  3-glucoside (mg/L)  =A×Mw×DF×1000ε×L,

where *A* is the difference of anthocyanin absorbance, in two different mediums and calculated as (*A*_510 nm_−*A*_700 nm_)_pH 1.0_ − (*A*_510 nm_−*A*_700 nm_)_pH 4.5_, *M*_*w*_ is the molecular weight of cyanidin 3-glucoside (= 449.2 g/mol), DF is dilution factor, *L* is the path length of cell (1 cm), *ε* is the molar absorptivity of cyanidin 3-glucoside (= 26900 lit/mol.cm), and 1000 indicates the conversion factor of gram to milligram [21].

### 2.3. Induction of Pancreatitis

Acute pancreatitis was induced by five intraperitoneal (i.p.) injection of 50 *μ*g/kg body weight of cerulein (Sigma, St. Louis, MO, USA) with 1 h intervals according to the method which was previously demonstrated by Mazzon et al. [22]. Cerulein causes edematous pancreatitis with prominent amylase and lipase level, increased TNF-alpha, myeloperoxidase (MPO) activity, and extensive lipid peroxidation. 

### 2.4. Animals

Male mice weighting 25–30 g and bred in animal house (Isfahan School of Pharmacy, Isfahan, Iran) were used in this study. Animals were kept in uniform environment of temperature, humidity, and light/dark cycles (12/12 h) and allowed free access to pelleted rodent chow and tap water. 

Before initiation the experiment animals were fasted over the night. The study was approved by the Ethics Committee for Animal Care and Uses, Isfahan University of Medical Sciences, Isfahan, Iran.

### 2.5. Grouping

Animals were randomly assigned into the following 5 groups (*n* = 6).

Sham group: normal mice pretreated with normal saline (5 mL/kg i.p.).

Negative control groups: mice with acute pancreatitis were pretreated with normal saline (5 mL/kg and i.p). 

EAE groups: mice with acute pancreatitis were pretreated with (100, 200, and 400 mg/kg) as a single dose (i.p.). Test doses of *E. amoenum* extracts were chosen because they were in the range of safe therapeutic doses reported by Mehrabani et al. and did not show signs of hepatotoxicity [23]. Applied hydroalcoholic extract was dispersed in normal saline solution as vehicle. 

Intraperitoneal treatments were carried out 0.5 h before pancreatitis induction. Mice were sacrificed 6 h after the last injection of cerulein. Blood samples were obtained by directed intracardiac puncture and stored at −70° for biochemical analysis. After decapitation, the pancreas was quickly removed and fixed in formaldehyde (10%) for histological examination. Besides, portions of this organ were promptly frozen in liquid nitrogen and stored at −70°C until assayed.

### 2.6. Amylase and Lipase Serum Level Analysis

Serum lipase and amylase activity were determined by using commercially available lipase and amylase kits (Pars-Azmoon Company, Tehran, Iran). 

### 2.7. Myeloperoxidase Activity Assay

MPO activity, an index of polymorphonuclear leukocyte accumulation, was measured according to the modified method of Bradley et al. [24]. Pancreas tissue was homogenized in 1 mL of 50 mM potassium phosphate buffer containing 0.5% HTAB (hexadecyltrimethylammonium bromide). Then, the homogenate was sonicated in an ice bath for 10 s, freeze-thawed thrice with sonication between cycles. After that, the suspensions were centrifuged at 15,000 rpm for 15 min at 4°C and then the supernatant (0.1 mL) was allowed to react with 2.9 mL of 50 mM potassium phosphate buffer (pH 6.0) containing O-dianisidine dihydrochloride (0.167 mg/mL) and 0.005% hydrogen peroxide. The absorbance of the reaction mixture was measured at 450 nm using a UV-Vis spectrophotometer. MPO activity was expressed in units (U) per gram of wet tissue weight.

### 2.8. Measurement of Lipid Peroxidation

The level of malondialdehyde (MDA) in pancreatic tissue was determined as an index of lipid peroxidation. Briefly 200 mL of tissue homogenate was added to 0.2 mL of 8.1% sodium dodecyl sulfate (SDS), 1.5 mL of 20% acetic acid (pH 3.5), 1.5 mL of 0.8% aqueous solution of thiobarbituric acid, and 600 *μ*L of distilled water. The mixture was heated at 95°C for 60 min and centrifuged at 4000 rpm for 10 min; its absorbance was then measured at 532 nm. The standard curve was obtained by 1,1,3,3-tetramethoxypropane and results were expressed as nanomoles of MDA per gram of wet tissue [25].

### 2.9. Measurement of Pancreatic Cytokines

Tissue TNF-alpha and IL-6 were measured using an enzyme-linked immunosorbent assay (ELISA) commercial kit according to the manufacturer's instructions (TNF-*α* ELISA kit Glory Science Co., Ltd., Hong Kong, IL-6 ELISA kit Boster Biological Technology, Ltd., China). The cytokine levels were calculated after plotting the standard curves and expressed as pg/mL.

### 2.10. Histological Examination

Paraffin-embedded pancreas samples were sectioned (5 *μ*m), stained with hematoxylin and eosin (H&E), and examined by coworker pathologist unaware from experimental protocol.

The histological grading of edema was made using a scale ranging from 0 to 3 (0 = no edema, 1 = interlobular edema, 2 = interlobular and moderate intralobular edema, and 3 = interlobular edema and severe intralobular edema). Leukocyte infiltration was also graded from 0 to 3 (0 = absent, 1 = scarce perivascular infiltration, 2 = moderate perivascular and scarce diffuse infiltration, and 3 = abundant diffuse infiltration). Grading of vacuolization was based on the appropriate percentage of acinar cells involved 0 = absent, 1 = less than 25%, 2 = 25–50%, and 3 = more than 50% of acinar cells [26].

### 2.11. Statistical Analysis

Biochemical results are expressed as mean ± SEM. Statistical analysis was carried out by one-way analysis of variance (ANOVA) followed by Tukey's multiple comparison test. Nonparametric data was analyzed by Mann-Whitney *U* test. The minimal level of significance was considered at *P* < 0.05.

## 3. Results

### 3.1. Anthocyanin Content

Anthocyanins of EAE was 3.1% of cyanidin-3-glucoside equivalent.

### 3.2. Effects of EAE on the Serum Levels of Amylase and Lipase

**Figure 1:**
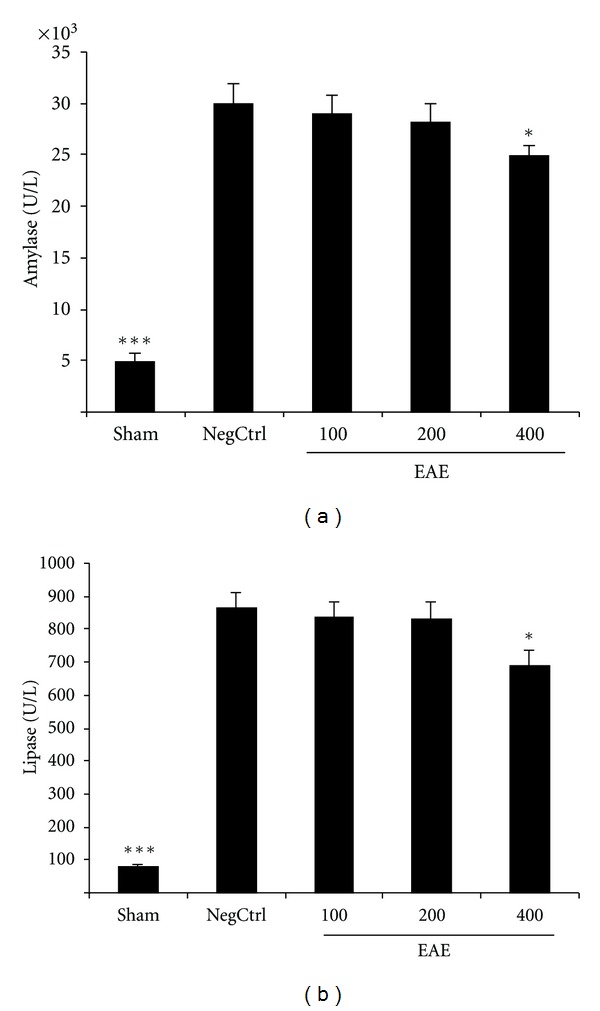
(a) Effect of EAE on serum amylase level (U/L) of cerulein-induced acute pancreatitis in mice. Sham: normal mice treated with normal saline (5 mL/kg), NegCtrl: negative control treated with normal saline (5 mL/kg), EAE: *E. amoenum* extract treated mice (100, 200, 400 mg/kg). Data are shown as means ± SEM of 6 animals for each group. *P < 0.05, ***P < 0.001 versus negative control (ANOVA). (b) Effect of EAE on serum lipase level (U/L) of cerulein-induced acute pancreatitis in mice. Sham: normal mice treated with normal saline (5 mL/kg), NegCtrl: negative control treated with normal saline (5 mL/kg), EAE: *E. amoenum* extract treated mice (100, 200, 400 mg/kg). Data are shown as means ± SEM of 6 animals for each group. *P < 0.05, ***P < 0.001 versus negative control (ANOVA).

Cerulein-induced pancreatitis in vehicle-treated mice was associated with significant rises in the serum levels of amylase and lipase. The increase in amylase and lipase was markedly reduced in cerulein-treated mice which had been pretreated with EAE in dose of 400 mg/kg by i.p. injection (Figures 1(a) and 1(b)).

### 3.3. Effects of EAE on Production of Proinflammatory Cytokines

**Figure 2:**
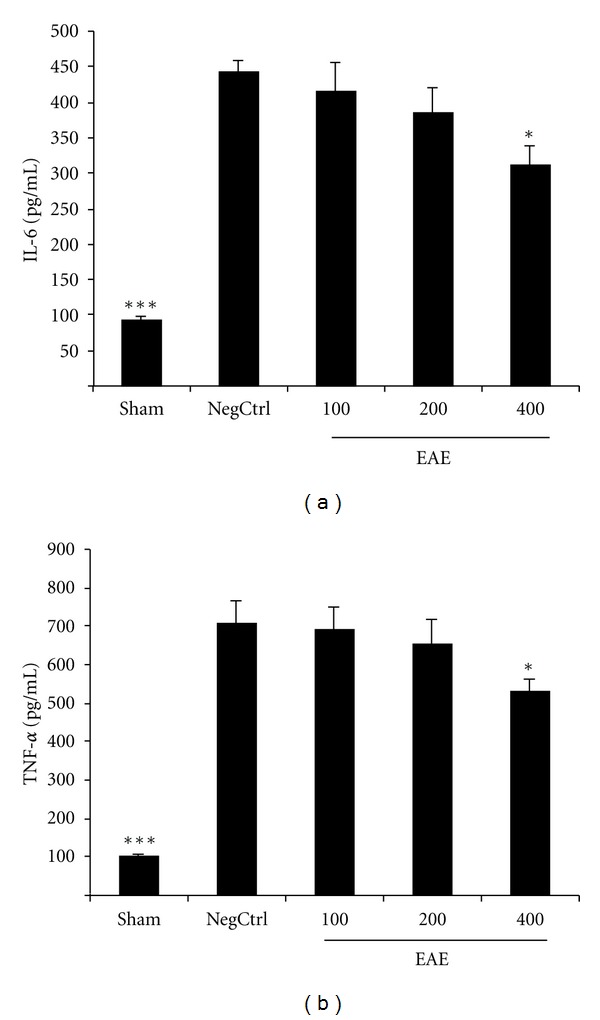
(a) Effect of EAE on pancreatic IL-6 level (pg/mL) of cerulein-induced acute pancreatitis in mice. Sham: normal mice treated with normal saline (5 mL/kg), NegCtrl: negative control treated with normal saline (5 mL/kg), EAE: *E. amoenum* extract treated mice (100, 200, 400 mg/kg). Data are shown as means ± SEM of 6 animals for each group. *P < 0.05, ***P < 0.001 versus negative control (ANOVA). (b) Effect of EAE on pancreatic TNF-alpha level (pg/mL) of cerulein-induced acute pancreatitis in mice. Sham: normal mice treated with normal saline (5 mL/kg), NegCtrl: negative control treated with normal saline (5 mL/kg), EAE: *E. amoenum* extract treated mice (100, 200, 400 mg/kg). Data are shown as means ± SEM of 6 animals for each group. *P < 0.05, ***P < 0.001 versus negative control (ANOVA).

Cerulein administration caused significant increase of IL-6 and TNF-alpha formation in vehicle-treated mice (negative control). Pancreas levels of IL-6 and TNF-alpha were significantly reduced (*P* < 0.05) in cerulein treated mice which had been pretreated with EAE in dose of 400 mg/kg by i.p. injection (Figures 2(a) and 2(b)).

### 3.4. Effects of EAE on MPO Activity

**Figure 3:**
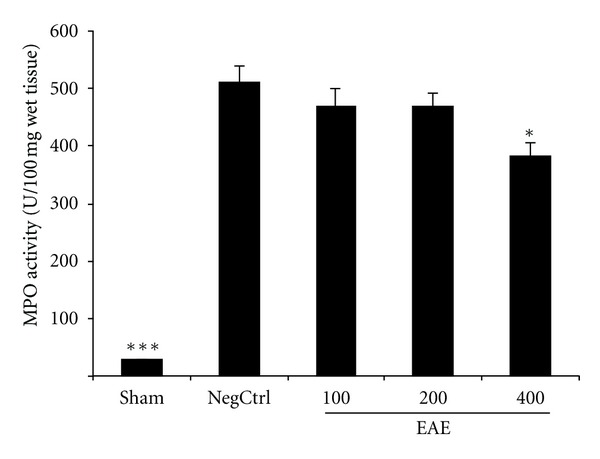
Effect of EAE on pancreatic MPO (myeloperoxidase) activity (U/g wet tissue) of cerulein-induced acute pancreatitis in mice. Sham: normal mice treated with normal saline (5 mL/kg), NegCtrl: negative control treated with normal saline (5 mL/kg), EAE: *E. amoenum* extract treated mice (100, 200, 400 mg/kg). Data are shown as means ± SEM of 6 animals for each group. *P < 0.05, ***P < 0.001 versus negative control (ANOVA).

MPO activity as a marker of leukocyte accumulation was obviously enhanced in the pancreas tissue following the cerulein administration. Pretreatment with EAE in dose of 400 mg/kg by i.p. injection significantly diminished MPO activity, in comparison to cerulein treated mice (*P* < 0.05) (Figure 3).

### 3.5. Effects of EAE on Lipid Peroxidation

**Figure 4:**
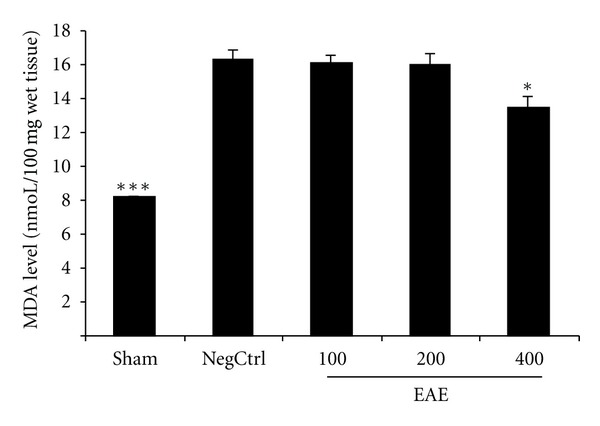
Effect of EAE on pancreatic MDA (malondialdehyde) level (nmoL/100 mg wet tissue) of cerulein-induced acute pancreatitis in mice. Sham: normal mice treated with normal saline (5 mL/kg), NegCtrl: negative control treated with normal saline (5 mL/kg), EAE: *E. amoenum* extract treated mice (100, 200, 400 mg/kg). Data are shown as means ± SEM of 6 animals for each group. *P < 0.05, ***P < 0.001 versus negative control (ANOVA).

The level of MDA as an index of lipid peroxidation was substantially increased in cerulein treated mice (negative control). MDA levels of Pancreatic tissue were obviously reduced by EAE pretreatment in dose of 400 mg/kg i.p. administration (*P* < 0.05) (Figure 4).

### 3.6. Effects of EAE on the Histological Parameters

In normal saline treated mice, pancreas did not show any tissue injuries at light microscopic level (×10 magnification). Administration of cerulein induced acute edematous with severe leukocyte infiltration pancreatitis in all mice tested. Prominent interlobular and severe intralobular edema was also accompanied with moderate perivascular and abundant diffuse inflammatory infiltration, but no vacuolization, necrosis, or hemorrhages were seen.

**Figure 5:**
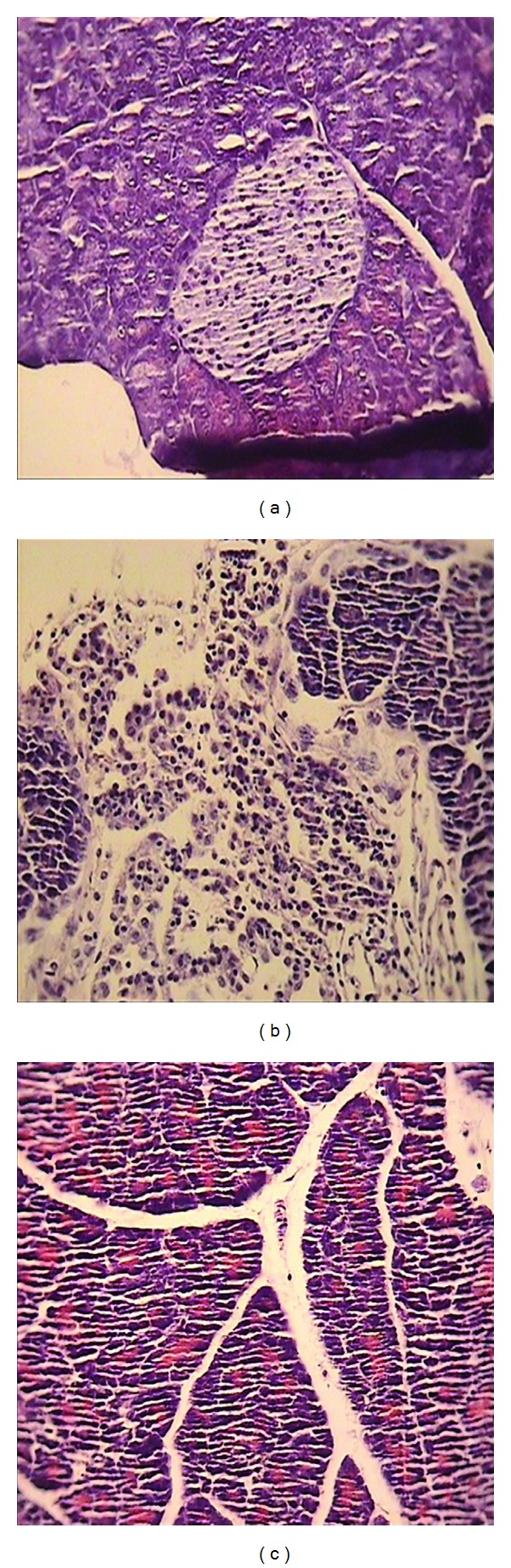
Representative illustration of normal pancreas and acute pancreatitis in mice. (a) Normal pancreatic tissue. (b) Acute pancreatitis induced by cerulein with severe intralobular edema and abundant diffuse infiltration. (c) Acute pancreatitis treated with EAE (400 mg/kg) with moderate intralobular edema and scarce perivascular infiltration. H&E staining with low (×10) power.

In groups that received extract in the dose of 400 mg/kg, the severity of edema and leukocyte infiltration significantly reduced compared to normal saline treated group (*P* < 0.05). (Table 1, Figure 5).

## 4. Discussion

In the present study, results showed that *E. amoenum* had good potential to attenuate pancreatitis in mice as indicated by biochemical, immunological, and histological evaluations. Biochemical and immunological assays confirmed that administration of EAE reduced amylase and lipase activity, TNF-alpha, IL-6, MPO activity, and lipid peroxidation which are markers of pancreatitis. Interestingly EAE, especially at the dose of 400 mg/kg i.p., showed significant protection against pancreatitis compared to control group. Regarding the histological results, administration of EAE showed an effective protection in a manner that was partly dependent to the dose because the highest dose of EAE (400 mg/kg i.p.) had significant effects compared to the control group while other two smaller doses were not effective

This finding was repeated in different examinations and the results showed that the lower doses of extract (100 and 200 mg/kg i.p.) were not effective to suppress pancreatitis and neither of doses had significant effects on serum levels of amylase and lipase activities as well as inflammatory mediators and lipid peroxidation. The dose of 400 mg/kg; however, was effective for all the parameters to be declined significantly. On the other hand, the difference between the applied doses was not statistically significant and this suggests that EAE protective activity on acute pancreatitis was at least partly dependent to the dose. This is in accordance with the results obtained by Zhao et al. [27] which demonstrated that the higher dose of rhubarb hydroalcoholic extract (150 mg/kg, twice daily, p.o.) was effective to protect against cerulein-induced acute pancreatitis while the lower test dose (75 mg/kg) was not effective.

Cerulein is an analog of cholecystokinin, which acts on CCK_1_ and CCK_2_ receptors and causes activation of the Jak2/Stat3 pathway [28]. The Jak/Stat pathway mediates a wide variety of biological effects, such as immune response, cell differentiation and proliferation, cell survival, or even oncogenesis [29]. On the other hand free oxygen radicals that generated from cerulein cause induction of oxidant-sensitive transcription factor (nuclear factor kappa-light-chain-enhancer of activated B cells, NF-*κ*B) activation thereby enhance cytokine expression and lipid peroxidation [30]. Therefore, any active component which inhibit or scavenge free radicals that liberated by cerulein could inhibit cytokine expression by suppression of NF-*κ*B activation and so reduce the inflammatory response associated with cerulein induced acute pancreatitis [31]. 

*E. amoenum* is considered as a promising source of bioactive compounds with various beneficial biological activities. Antibacterial, antioxidant, analgesic, anxiolytic, antidepressant, and immunomodulatory actions are the most important properties of *E. amoenum* [7–12].

The antioxidant activity of Iranian *E. amoenum* flower aqueous extract has been investigated by Ranjbar et al. in human. The results showed a significant reduction in blood lipid peroxidation after 14 days of extract (7 mg/kgs) intake. They suggested that this antioxidant potential of *E. amoenum* may be due to its bioactive components, especially its flavonoids [13]. 

Flavonoids with anti-inflammatory, antioxidant, and gastroprotective effects are widely distributed in plant kingdom [32]. Stimulation of prostaglandins, suppression of histamine secretion, and inhibition of *Helicobacter pylori* growth are implicated for the gastroprotective effects of flavonoids [33]. 

It could be suggested that EAE exerted its protective effects through mechanisms that are not essentially dependent to phenolic contents of extract and antioxidant effects. Indeed the presence of some bioactive compounds like rosmarinic acid with anti-inflammatory effect [34] and Cyanidin 3-glucoside that inhibits the activation and translocation of c-Jun and NF-*κ*B factors into nucleus suggests that suppression of cyclooxygenase-2 expressions [15] and reduced intracellular reactive oxygen species (ROS) levels via activating the glutathione (GSH) antioxidant system [16] might be involved. Delphinidin which inhibits TNF-alpha-induced COX-2 expression by directly inhibiting Fyn kinase activity [17] is one of the other active compounds which could be found in EAE and proposed as the other mechanisms in which it may be involved in protective effect of EAE on cerulein-induced acute pancreatitis in mice.

Administration of medicinal herbs that possess anti-inflammatory and antioxidant properties is a new approach to attenuate inflammatory-related disorders [35]. Our previous study showed that *Cichorium intybus* L. hydroalcoholic extracts possessed protection against cerulein-induced acute pancreatitis in mice [36]. Similarly, in this regard effects of *Ginkgo biloba* extract on acute pancreatitis have been studied by Zeybek et al. [37]. The results demonstrated that *G. biloba* extract at 100 mg/kg administered i.p. was able to decrease significantly in serum amylase and lipase levels as well as histopathologic scores in sodium taurocholate-induced pancreatitis. The beneficial effects had attributed to the oxygen radical scavenging potential of *G. biloba* flavonoids contents. Our findings indicated that various mechanisms were involved in protective activity of EAE on pancreatitis because biochemical, immunological, and histological parameters were improved. So it is plausible to accept that different active compounds of applied extract were responsible for these effects. Thus further experimental studies are necessary to isolate and identify the active principles present in *E. amoenum* fractions which are responsible for the protective effects on pancreatitis. 

## 5. Conclusion

We demonstrated that *E. amoenum* hydroalcoholic extract possessed protective activity against cerulein-induced acute pancreatitis in mice and may suggest a therapeutic potential for therapy or prevention in this inflammatory disease condition.

## Figures and Tables

**Table 1:** Effect of EAE extract on pathological scores of pancreas in cerulein-induced acute pancreatitis in mice.

Group	Route	Edema	Leukocyte infiltration
Sham	i.p.	0.0	0.0
NegCtrl	i.p.	2.6 ± 0.2	2.6 ± 0.2
EAE 100	i.p.	2.1 + 0.3	2.3 ± 0.3
EAE 200	i.p.	2.0 ± 0.3	2.1 ± 0.3
EAE 400	i.p.	1.3 ± 0.2*	1.5 ± 0.2*

Sham: normal mice treated with normal saline, NegCtrl: negative control treated with normal saline, EAE: *E. amoenum* extract treated mice (100, 200, 400 mg/kg).

Data are shown as means ± SEM, *n* = 6 (Mann-Whitney *U* test).

*P < 0.05: significant difference compared to negative control group.
